# Mutation of *hmgA*, encoding homogentisate 1,2-dioxygenase, is responsible for pyomelanin production but does not impact the virulence of *Burkholderia cenocepacia* in a chronic granulomatous disease mouse lung infection

**DOI:** 10.1128/spectrum.00410-24

**Published:** 2024-05-29

**Authors:** Dina A. Moustafa, Linda Wu, Melissa Ivey, Sarah C. Fankhauser, Joanna B. Goldberg

**Affiliations:** 1 Division of Pulmonary, Asthma, Cystic Fibrosis, and Sleep, Department of Pediatrics, Emory University School of Medicine, Atlanta, Georgia, USA; 2 Department of Biology, Oxford College of Emory University, Oxford, Georgia, USA; University of Manitoba, Winnipeg, Manitoba, Canada

**Keywords:** *Burkholderia*, pyomelanin, pigment, CGD

## Abstract

The *Burkholderia cepacia* complex (Bcc) is a group of Gram-negative opportunistic bacteria often associated with fatal pulmonary infections in patients with impaired immunity, particularly those with cystic fibrosis (CF) and chronic granulomatous disease (CGD). Some Bcc strains are known to naturally produce pyomelanin, a brown melanin-like pigment known for scavenging free radicals; pigment production has been reported to enable Bcc strains to overcome the host cell oxidative burst. In this work, we investigated the role of pyomelanin in resistance to oxidative stress and virulence in strains J2315 and K56-2, two epidemic CF isolates belonging to the *Burkholderia cenocepacia* ET-12 lineage. We previously reported that a single amino acid change from glycine to arginine at residue 378 in homogentisate 1,2-dioxygenase (HmgA) affects the pigment production phenotype: pigmented J2315 has an arginine at position 378, while non-pigmented K56-2 has a glycine at this position. Herein, we performed allelic exchange to generate isogenic non-pigmented and pigmented strains of J2315 and K56-2, respectively, and tested these to determine whether pyomelanin contributes to the protection against oxidative stress *in vitro* as well as in a respiratory infection in CGD mice *in vivo*. Our results indicate that the altered pigment phenotype does not significantly impact these strains’ ability to resist oxidative stress with H_2_O_2_ and NO *in vitro* and did not change the virulence and infection outcome in CGD mice *in vivo* suggesting that other factors besides pyomelanin are contributing to the pathophysiology of these strains.

**IMPORTANCE:**

The *Burkholderia cepacia* complex (Bcc) is a group of Gram-negative opportunistic bacteria that are often associated with fatal pulmonary infections in patients with impaired immunity, particularly those with cystic fibrosis and chronic granulomatous disease (CGD). Some Bcc strains are known to naturally produce pyomelanin, a brown melanin-like pigment known for scavenging free radicals and overcoming the host cell oxidative burst. We investigated the role of pyomelanin in *Burkholderia cenocepacia* strains J2315 (pigmented) and K56-2 (non-pigmented) and performed allelic exchange to generate isogenic non-pigmented and pigmented strains, respectively. Our results indicate that the altered pigment phenotype does not significantly impact these strains’ ability to resist H_2_O_2_ or NO *in vitro* and did not alter the outcome of a respiratory infection in CGD mice *in vivo*. These results suggest that pyomelanin may not always constitute a virulence factor and suggest that other features are contributing to the pathophysiology of these strains.

## INTRODUCTION

Cystic fibrosis (CF) and chronic granulomatous disease (CGD) are hereditary diseases characterized by inflammatory complications and recurrent infections with opportunistic bacterial and fungal pathogens (1, 2). Members of the *Burkholderia cepacia* complex (Bcc), a group of at least 23 genetically and closely related bacterial species (3) that rarely cause disease in healthy individuals and are widely distributed in the natural environment, are among the select pathogens that infect both CF and CGD patients. Bcc is especially troublesome due to its inherent antibiotic resistance, capacity to survive in supposedly sterile hospital solutions, and ability to spread between patients leading to worse outcome (4–7). These infections can also result in fatal necrotizing pneumonia and septicemia known as “cepacia syndrome” (8).

Bcc is known to produce a multitude of virulence factors that enhance their pathogenicity in different stages of infection in host cells. Bcc has been shown to survive intracellularly in both professional and nonprofessional phagocytes. In particular, they have been detected inside airway and alveolar epithelial cells, neutrophils, and in pulmonary macrophages from resected lungs of CF patients undergoing transplant (9, 10). Interestingly, in macrophages, some Bcc isolates can survive and develop an intracellular niche and evade host immune killing using several mechanisms against oxidative stress, such as interfering with phagosome maturation and phago-lysosomal fusion (11), and delaying the phagosome acidification (12) leading to their resistance to oxidative damage, which acts as an important survival strategy for Bcc inside the host. The intracellular survival of Bcc within macrophages has been previously studied and documented *in vitro* in various models (11, 13, 14). The presence of the melanin-like pigment, known as pyomelanin, has been proposed to have scavenging properties and protect the bacteria during the respiratory burst (12).

Melanin constitutes a general class of complex high-molecular-weight polyphenolic heteropolymers that include eumelanin, pheomelanin, and pyomelanin. Melanin is capable of serving as an electron trap for reactive oxygen species due to its ability to exist in multiple oxidation states (15). Pyomelanin is a pigment that is commonly found in many systems of life, particularly bacteria and fungi. The pigment has been reported to efficiently scavenge reactive oxygen species and diminish the oxidizing stress of the host due to its high tolerance to hydrogen peroxide, thus promoting the survival of these pigmented organisms in their host (16).

We have noted a subset of Bcc isolates can produce pyomelanin under normal laboratory conditions (15). In previous *in vitro* studies of others, pyomelanin has been demonstrated to contribute to the increased resistance of *B. cenocepacia* strain C5424 to extracellular hydrogen peroxide, which is the main type of reactive oxygen species found in macrophages (13). In that same study, a non-pigmented isogenic mutant of C5424 was found to exhibit a higher sensitivity to reactive oxygen species killing. While potentially advantageous, as mentioned, the pyomelanin production phenotype is not found in every member of Bcc; in particular, it is present in *B. cenocepacia* J2315 and absent in *B. cenocepacia* K56-2, two closely related strains of the ET-12 lineage, which were both isolated from CF patients, calling into question the potential role of pyomelanin in infection.

Pyomelanin is a natural polymer of homogentisic acid (HGA, 2,5-dihydroxyphenylacetic acid) synthesized through the L-tyrosine pathway, specifically due to the accumulation of HGA, which is synthesized via 4-hydroxyphenylpyruvate dioxygenase (4-HDDP). HGA is excreted and spontaneously auto-oxidizes to form benzoquinoneacetic acid, followed by self-polymerization to produce pyomelanin (15). Two essential enzymes in the synthesis of pyomelanin are HppD and HmgA. The *hppD* gene codes for a protein that is responsible for the conversion of 4-hydroxyphenylpyruvate to HGA. Homogentisate 1,2-dioxygenase, encoded by the *hmgA* gene, converts HGA to maleylacetoacetate. Our lab has previously reported that a single amino acid change from glycine (G; non-pigmented phenotype) to arginine (R; pigmented phenotype) at residue 378 of HmgA contributes to the pigment production phenotype. The G378R change renders HmgA non-functional, and the pathway stops at the intermediate molecule HGA, which ultimately results in pyomelanin production (15). While numerous studies had investigated the role of bacterial pyomelanin *in vitro*, particularly by deleting *hppD* or *hmgA* or complementing mutants (13, 16–20), crucially, there has been a gap in our understanding of the role of *B. cenocepacia* pyomelanin pigment in *in vivo* models that mimic human infection. We previously noted that J2315 has an R at position 378 of HmgA, while K56-2 has a G at this position. Our previous studies used *trans* complementation with a plasmid-borne wild-type copy of the *hmgA* gene from *B. cenocepacia* J2315 (pigmented) or K56-2 (non-pigmented); the resulting strains contained both the wild-type and a mutant copy of the *hmgA* gene. The altered pigmented phenotype of either strain did not confer any changes in the susceptibility to hydrogen peroxide (15). Here, we performed allelic exchange to generate isogenic non-pigmented and pigmented strains of J2315 and K56-2, respectively, and tested these to determine whether pyomelanin contributes to the protection of pigmented strains against oxidative stress *in vitro* as well as in a respiratory infection in CGD mice.

## MATERIALS AND METHODS

### Construction of *hmgA* mutants

*B. cenocepacia* J2315 and K56-2 were used as the parental strains to generate *hmgA* mutants. For cloning and conjugations, we used *Escherichia coli* DH5α and SM10, respectively. The *hmgA* gene was amplified from J2315 and K56-2 using the following primers: *hmgA*-F (5′ GGggtaccATGACGCTTGACCTGTCGAAAC 3′); *hmgA*-R (5′ ggaagcttTCATCGTTGCTCCGGATTGAAG 3′). The products were digested with KpnI and HindIII and ligated into similarly digested pEX18-Tc to generate pEX18-Tc::J*hmgA* and pEX18-Tc::K*hmgA*, respectively, and were electroporated into *E. coli* DH5α (21). Verified plasmids were then introduced into SM10 by electroporation. The donor SM10 strains containing the pEX18-Tc::J*hmgA* or pEX18-Tc::K*hmgA* plasmids were used for conjugations into K56-2 or J2315, respectively. For this, a mixture containing 100 µL of donor and 100 µL recipient was incubated on tryptic soy agar plates at room temperature for 1 hour and then moved to 37°C for 6 hours. Cells were resuspended and transconjugants were selected by plating on LB plates supplemented with 25 µg/mL of spectinomycin (to select against *E. coli*) and 300 µg/mL of tetracycline and incubated at 37°C for 3 days. Transconjugants were streaked on LB plates supplemented with 5% sucrose and incubated for 3 days at 37°C. Individual colonies were purified from the plates and tested for tetracycline sensitivity. Selected colonies were then analyzed for the correct *hmgA* integration by PCR and differential digest with AvaII.

### Comparing growth and pigment production between strains

Three milliliters cultures of all four strains were grown shaking overnight at 37°C in Luria broth (LB) medium. The next day strains were back diluted to an (optical density) OD_600_ of 0.01 into 50 mL of LB media. Cultures were incubated at 37°C shaking, and measurements of the culture at OD_600_ and cell-free supernatants at OD_480_ [for growth and pigment production (15), respectively] were taken at various times, as indicated.

### *In vitro* sensitivity and survival in the presence of reactive oxygen and nitrogen species

To test cultures for sensitivity to reactive oxygen and nitrogen species, bacteria were grown overnight at 37°C, shaking in LB medium. Late stationary phase cultures were back diluted to an OD_600_ of 0.2 and were incubated with various concentrations (1 mM to 200 mM) of the reactive oxygen species, hydrogen peroxide (H_2_O_2_) (Sigma-Aldrich), or the nitric oxide (NO) donor (16), sodium nitrosprusside (SNP) (Chem-ImpexInt’l Inc), in 96-well plates at 37°C for 18–20 hours. OD_600_ readings were taken every 20 minutes using a microplate reader (BioTek Instruments, Inc). The strains were incubated in duplicates, and the growth analysis was performed at each concentration of H_2_O_2_ and SNP.

To determine the survival in the presence of H_2_O_2_ and SNP, bacteria were treated with high concentrations of these species and plated for survival at fixed endpoints. For this, cultures were grown until late stationary phase and back diluted to an OD_600_ of 1.0. Strains were treated with 100 mM of either H_2_O_2_ or SNP. Aliquots were removed at 0-, 30-, 60-, and 120 minutes post-treatment, were serially diluted, and plated in duplicate on LB agar plates and incubated up to 48 hours at 37°C and counted for colony-forming units (CFU/mL). As a negative control, parallel untreated cultures of each strain were included, and they were aliquoted at the same indicated time points. The percent survival was calculated by dividing the CFU/mL at indicated fixed timepoints by parallel untreated cultures of each strain collected at the same timepoint.

### CGD mouse lung infections

This animal study was conducted according to the guidelines of the Emory University Institutional Animal Care and Use Committee (IACUC), under approved protocol number PROTO201700441. The study was carried out according to the established guidelines and policies at Emory University School of Medicine, and recommendations in the Guide for Care and Use of Laboratory Animals, and local, state, and federal laws.

*B. cenocepacia* J2315 and K56-2 and isogenic mutants were grown from glycerol stocks on LB agar plates, and then a single colony was grown in LB broth and then incubated with shaking at 37°C for 4–5 hours until an OD_600_ of 0.08 was reached. Cultures were spun down and washed twice with phosphate-buffered saline (PBS). Inoculum was adjusted spectrophotometrically to obtain the desired challenge dose of 1 × 10^3^ in a volume of 50 µL.

Mice deficient in the gp91phox subunit of the NADPH oxidase [B6.129S6-Cybbtm1Din/J (gp91^phox−/−^); Jackson Laboratory, Bar Harbor, ME] were bred and maintained at the Emory Division of Animal Resources (DAR). Eight- to 10-week-old animals gender matched were anesthetized by intraperitoneal injection of 0.2 mL of ketamine (10  mg/mL) and xylazine (0.5  mg/mL). Mice were infected by non-invasive intratracheal instillation of J2315 and K56-2 or isogenic mutant strains, as previously described (22). For colonization experiments, mice were euthanized at indicated time points, and whole lungs were collected aseptically and homogenized in 1 mL PBS. Lung homogenates were serially diluted and plated onto *Pseudomonas* isolation agar (BD Difco) for CFU determination. For survival experiments, mice were monitored up to 12 days post-infection, and animals that succumbed to infection or appeared to be under acute distress were humanely euthanized and were included in the reported results.

## RESULTS

### *B. cenocepacia* J2315 and K56-2 show distinct levels of virulence in a CGD mouse lung infection model

**Fig 1:**
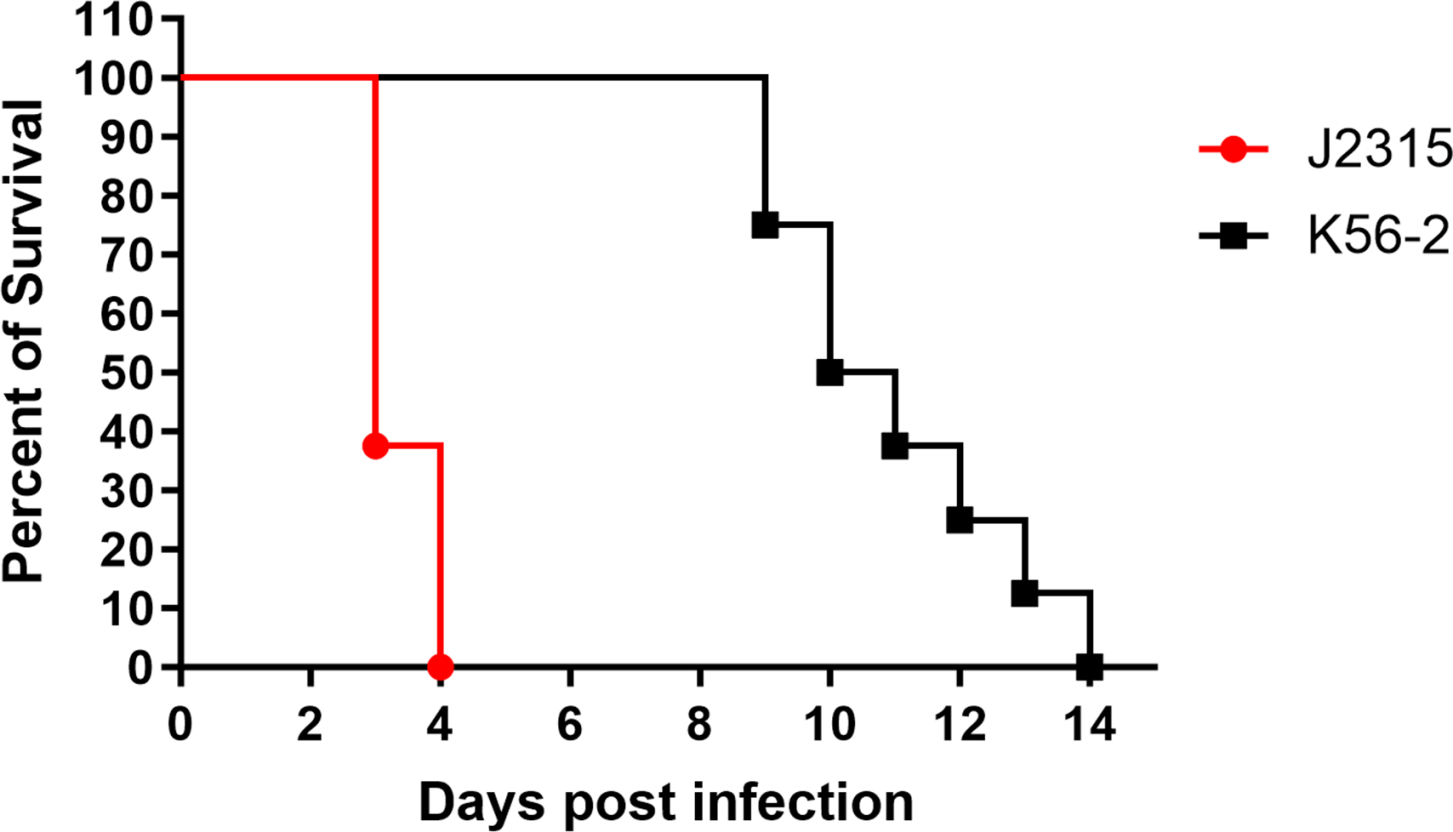
*B. cenocepacia* J2315 and K56-2 show distinct levels of virulence in a CGD mouse lung infection model. gp91^phox−/−^ (CGD) mice were infected intratracheally with ∼10^3^ CFU/mouse of strains J2315 and K56-2. Mice were monitored for 14  days post-infection. Results are represented by Kaplan-Meier survival curves and were analyzed by log rank test. Results were compiled from two independent experiments, *n* = 8/strain.

The ET-12 lineage *B. cenocepacia* strains, J2315 and K56-2, were tested for virulence in a respiratory infection model in CGD mice. We used gp91^phox−/−^ mice and delivered 1 × 10^3^ CFU of bacteria intratracheally. As seen in Fig. 1, all CGD mice infected with J2315 succumbed by day 4 post-infection. On the other hand, mice infected with K56-2 showed delayed time to death and animals started to succumb to infection starting at day 9 post-infection. These results indicate the J2315 is more virulent than K56-2 in this model of lung infection.

### A single amino acid change in HmgA is sufficient for pyomelanin production in *B. cenocepacia*

Our lab has previously reported that a single amino acid change from glycine to arginine at residue 378 of HmgA contributes to the pigmented phenotype. An arginine at residue 378 renders the HmgA protein nonfunctional and the pathway stops at the intermediate molecule homogentisic acid (HGA), which ultimately results in pyomelanin production. We found that J2315 had an arginine at position 378, while K56-2 had an glycine (15).

**Fig 2:**
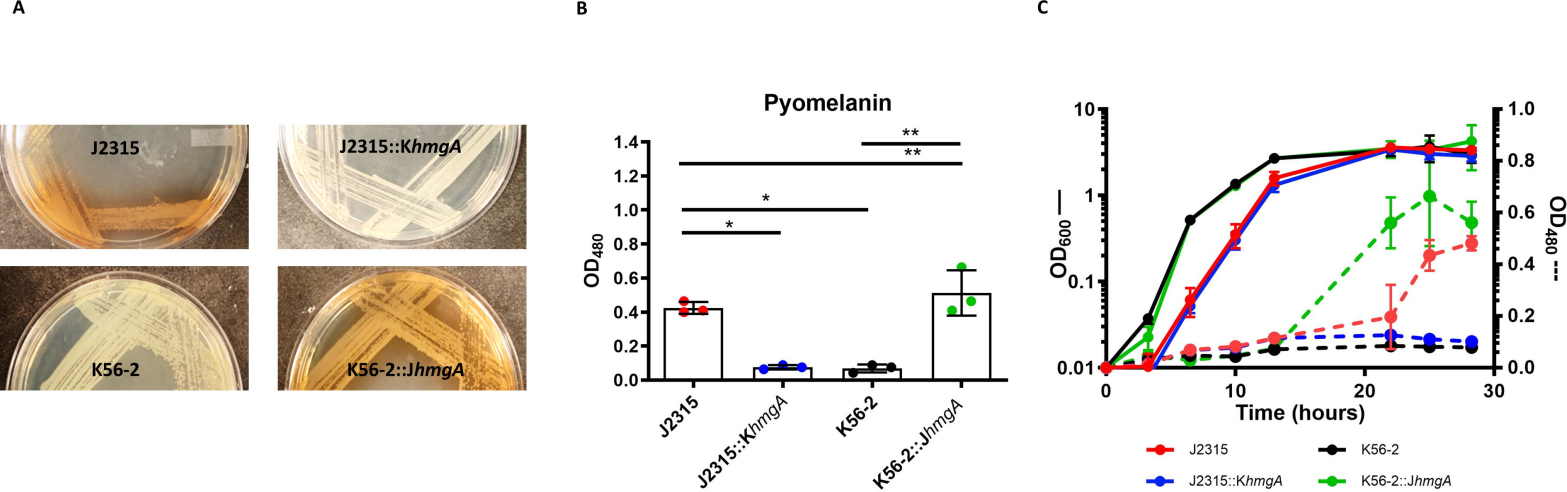
A single amino acid change in HmgA is sufficient for pyomelanin production in *B. cenocepacia*. (**A**) Colony phenotypes of wild-type J2315 (top) and K56-2 (bottom) and the designated isogenic mutants on LB agar media. Strains were incubated at 37°C for 48 hours. (**B**) Pyomelanin production in culture supernatant grown for 28 hours in LB. Cultures were normalized to an OD_600_ of 0.1, and supernatant was measured by OD_480_. (**C**) Growth and pigment production of all four strains incubated with shaking at 37°C in LB cultures. Growth was measured by OD_600_ on the left *Y* axis, pyomelanin production measured by OD_480_ on the right *Y* axis. Solid lines represent OD_600_ measurements and dashed lines represent OD_480_ measurements. For culture experiments, each point represents the mean of three independent cultures and error bars represent standard deviation. Data were analyzed by one-way ANOVA. *P < 0.05, **P < 0.01 by one-way ANOVA. Unlabeled comparisons were not statistically significant.

To determine whether this amino acid substitution in HmgA was sufficient for pyomelanin production in *B. cenocepacia*, we used allelic exchange to construct a J2315 strain containing the *hmgA* from K56-2 and a K56-2 strain containing the *hmgA* from J2315. This gave us two pairs of isogenic strains (J2315 and J2315::K*hmgA* as well as K56-2 and K56-2::J*hmgA*), which we used to determine the role of pyomelanin. After 48 hours of growth on solid LB media, J2315::K*hmgA* did not produce visible pigment, as compared to J2315, while K56-2::J*hmgA* did produce pigment as compared to K56-2 (Fig. 2A). We observed the same phenotypes of pyomelanin production as measured by the absorbance of the culture supernatant of all strains grown for 28 hours in liquid LB media (Fig. 2B).

To determine whether pyomelanin production would alter growth under these conditions, we performed growth curves in LB with each of the four strains. Over nearly 30 hours, there was no difference in the growth rate between the isogenic strains (K56-2 vs K56-2::J*hmgA* or J2315 vs J2315::K*hmgA)*. However, we did note that the J2315 strains grew slower than the K56-2 strains under these conditions (Fig. 2C). When we monitored pigment production during the course of this experiments, no pigment was produced by K56-2 or J2315::K*hmgA*, while K56-2::J*hmgA* and J2315 both produced pigment as detected in the supernatant, with K56-2::J*hmgA* producing it between 12 and 24 hours and J2315 producing it after 24 hours (Fig. 2C).

These results demonstrate the functionality of HmgA, and by extension pigment production, does not alter growth in LB. These data also indicate that the production of pyomelanin in J2315 does not account for its decreased growth rate compared to K56-2. Finally, these results show that a single amino acid change (from glycine to arginine) in HmgA is responsible for the production of pigment in these strains.

### Effect of pyomelanin production in protection against specific immune defense molecules *in vitro*

**Fig 3:**
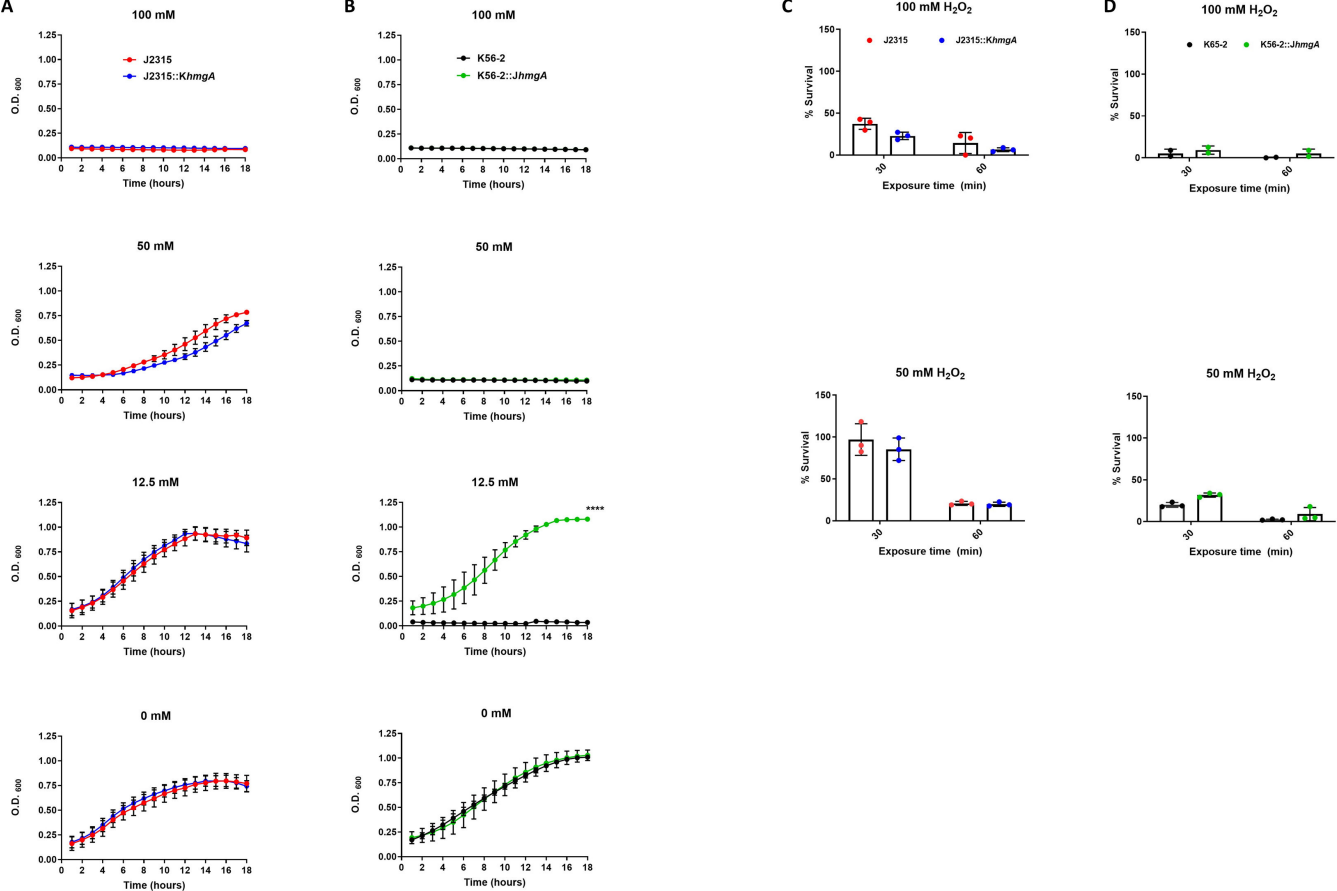
Pyomelanin production and *in vitro* susceptibility to killing by hydrogen peroxide. Growth curves of wild-type and designated isogenic mutant of (**A**) J2315 and (**B**) K56-2 treated with various concentrations of hydrogen peroxide (H_2_O_2_). Stationary phase cultures were back diluted to an OD_600_ = 0.2 and were incubated with indicated concentrations of H_2_O_2._ Viable CFU counts of wild-type and isogenic mutants of (**C**) J2315 or (**D**) K56-2 were determined after treatment with indicated H_2_O_2_ concentrations for 30 or 60 minutes. For panels (**C**) and (**D**), the percent survival was calculated by dividing the CFU/mL at fixed timepoints (30 or 60 minutes) by parallel untreated cultures of each strain. For all experiments, the data are pooled from three independent experiments. Error bars represent standard deviation (SD). Data were analyzed by student’s *t* test. ****P < 0.0001. Unlabeled comparisons were not statistically significant.

Previous work by Keith et al. (13) showed in *B. cenocepacia* C5424 that pyomelanin is associated with increased resistance to reactive oxygen species, like hydrogen peroxide (H_2_O_2_). However, whether this is a strain-specific phenomenon is unclear. To begin to determine whether pyomelanin production would alter resistance characteristics to specific immune response molecules in J2315 and K56-2, we performed similar studies on our strains. We monitored growth over 16 hours in various concentrations of H_2_O_2_ (0–100 mM). J2315 was more resistant to this treatment than K56-2. We found that J2315 showed growth inhibition at 100 mM, while K56-2 showed growth inhibition at 50 mM. However, the absence of pyomelanin did not seem to affect the resistance of J2315 mutant to H_2_O_2_, as the growth of J2315::K*hmgA* strain, under these conditions, was not altered compared to wild-type J2315 strain (Fig. 3A), which suggest that in this strain pyomelanin pigment is dispensable and other factors are playing a major role in the resistance of J2315 to H_2_O_2_. On the other hand, while the growth of the non-pigmented wild-type K56-2 strain was abolished at a concentration of 12.5 mM H_2_O_2_, K56-2::J*hmgA* showed increased growth at the same H_2_O_2_ concentration (Fig. 3B). These results suggest that under these experimental conditions, pyomelanin pigment is contributing to the resistance of K56-2::J*hmgA* to H_2_O_2_.

To determine the impact of pyomelanin on the survival of the strains after short-term exposure to reactive oxygen, we treated the strains with high doses of H_2_O_2_ (50 and 100 mM) and plated for survival at fixed timepoints (30 and 60 minutes) post-exposure. There was a modest decrease in survival of the non-pigmented strains, but these differences were not statistically significant (Fig. 3C and D).

**Fig 4:**
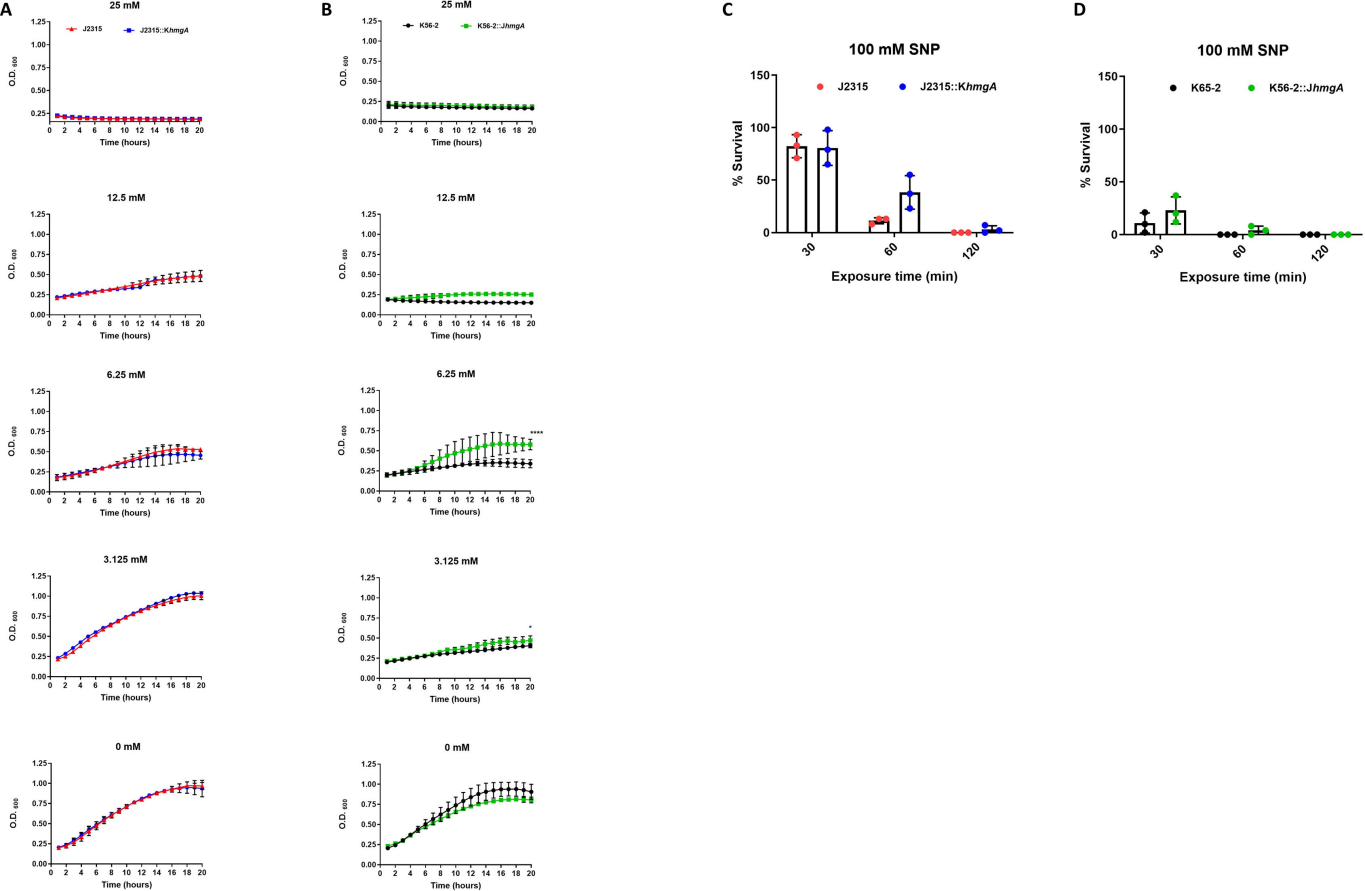
Effect of pyomelanin production in protection against reactive nitrogen species *in vitro*. Growth curves of wild-type and designated isogenic mutant of (**A**) J2315 and (**B**) K56-2 treated with various concentrations of sodium nitroprusside (SNP). Stationary phase cultures were back diluted to an OD_600_ = 0.2 and were incubated with indicated concentrations of SNP. Viable CFU counts of wild-type and isogenic mutants of (**C**) J2315 and (**D**) K56-2 were determined after treatment with indicated SNP concentrations for 30, 60, or 120 minutes. For panels (**C**) and (**D**), the percent survival was calculated by dividing the CFU/mL at fixed timepoints (30, 60, or 120 minutes) by parallel untreated cultures of each strain. For all experiments, the data are pooled from three independent experiments. Error bars represent standard deviation (SD). Data were analyzed by student’s *t* test. *P < 0.05, ****P < 0.0001. Unlabeled comparisons were not statistically significant.

In order to test pyomelanin’s role in resistance to reactive nitrogen species, we exposed all four strains to sodium nitroprusside (SNP), a nitric oxide (NO) donor, and measured their growth over 20 hours. Similar to what we observed for the H_2_O_2_ treatment, we noted that J2315 was more resistant to SNP than K56-2, which was particularly evident at 12.5 mM SNP, comparing results in Fig. 4A and B. Additionally, we observed that nonpigmented J2315::K*hmgA* demonstrated similar growth at the various concentrations of SNP compared to wild-type J2315 (Fig. 4A). This suggests that both the pigmented and non-pigmented phenotypes of J2315 were equally resistant to the NO generated by SNP. Conversely, we noted that at a SNP concentration of 6.25 mM, pigmented K56-2::J*hmgA* demonstrated higher growth compared to nonpigmented wild-type K56-2 (Fig. 4B). These results suggest a potential protective role of pyomelanin pigment under these experimental conditions in K56-2.

When we treated cultures of bacteria with 100 mM SNP for 2 hours, K56-2::J*hmgA* showed a higher survival than K56-2 (Fig. 4D) although this difference was not statistically significant. Distinct from this were the results with the isogenic J2315 strains where J2315::K*hmgA* demonstrated a higher survival compared to J2315 (Fig. 4C). This result suggests that there may be an advantage of the pigment *in vitro* to protect against reactive nitrogen species in the K56-2 strain background; however, since J2315::K*hmgA* survives much better than K56-2, this suggests there are additional strain-specific defenses against reactive nitrogen species in J2315 (Fig. 4C and D).

### HmgA does not alter either the survival or the colonization in a CGD mouse lung infection

**Fig 5:**
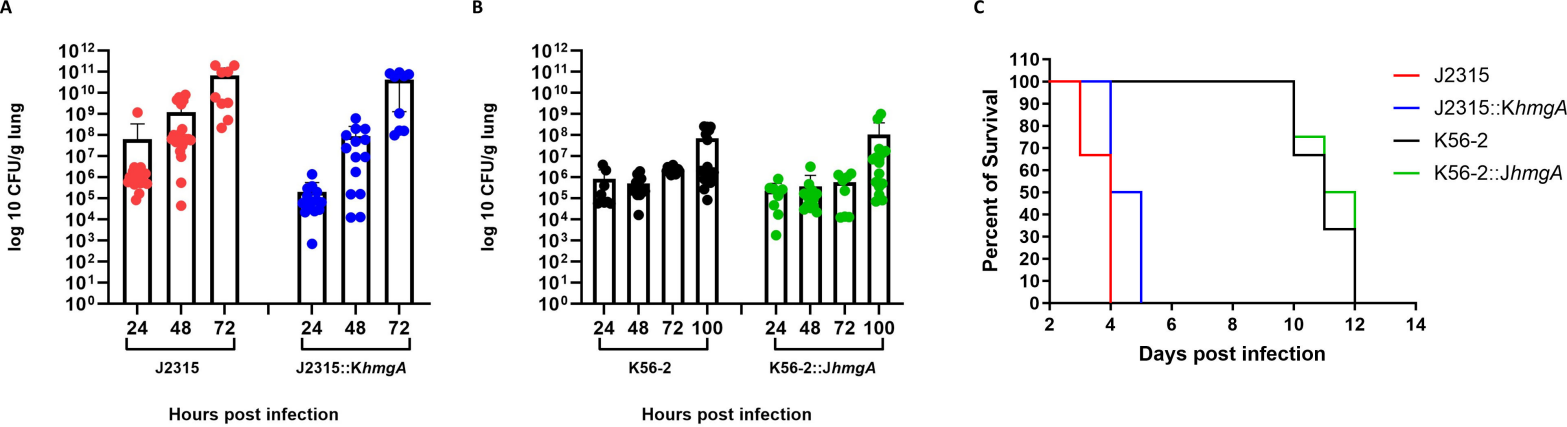
Altered pigment production does not impact the virulence of J2315 or K56-2 in a CGD mouse lung infection. Mice were infected intratracheally with ∼10^3^ CFU/mouse and the bacterial load in lung homogenates of mice infected with the wild-type or indicated isogenic mutants of (**A**) J2315 or (**B**) K6-2 was assessed. (**C**) A parallel group of mice were monitored for survival. For colonization experiments, mice were euthanized at indicated time points post-infection, and lungs were aseptically collected and homogenized in 1 mL PBS. All samples were plated for viable CFU on *Pseudomonas* isolation agar. Each dot represents the CFU from a single mouse. Error bars represent standard deviation (SD). Results were compiled from two independent experiments. For survival experiments, animals were monitored for 12 days post-infection. Results are represented by Kaplan-Meier survival curves and were analyzed by log rank test. Results were compiled from two independent experiments, *n* = 8/strain. Unlabeled comparisons were not statistically significant.

In order to determine whether pyomelanin plays a role during respiratory infection in CGD mice, we performed a non-invasive intratracheal infection of 10^3^ CFU/mL of each strain into CGD mice lungs. After the infection, we harvested lung tissue at fixed time-points post-infection, and we observed no significant difference between the colonization level of J2315 and J2315::K*hmgA* (Fig. 5A), as well as between K56-2 and K56-2::J*hmgA* (Fig. 5B).

A parallel group of infected mice were monitored for survival. Our results indicate that the absence of the pigment in J2315::K*hmgA* did not seem to compromise the virulence of this strain. Mice infected with either J2315 and its non-pigmented mutant J2315::K*hmgA* succumbed to infection starting at day 4 post-infection (Fig. 5C). Similarly, mice infected with K56-2 or its pigmented mutant K56-2::J*hmgA* showed similar time to death as all mice succumbed to infection starting at day 9 post-infection (Fig. 5C). As the wild-type strains and their isogenic mutants showed no statistically significant difference in the killing of CGD mice, these results suggest a very minor, if any, contribution of HmgA function to virulence in this model of infection.

## DISCUSSION

*B. cenocepacia* J2315 was isolated from a CF patient in Edinburgh in 1989 who was the index case for a patient-to-patient outbreak that spread to Toronto (4). *B. cenocepacia* K56-2 was isolated from a CF patient in Toronto (23). These isolates belong to the same clonal complex, the ET-12 lineage (24). J2315 was the first *B. cenocepacia* strain sequenced (25), while K56-2 has been used more extensively for molecular genetic studies because of its high transformation rate (26). Interestingly, previous studies have shown that these two strains are distinct in their virulence. Specifically, various *in vitro* models such as zebrafish (27), *Caenorhabditis elegans* (28), *Galleria mellonella* (29), and alfalfa (30) showed that K56-2 was more virulent than J2315. However, whether J2315 and K56-2 would show differential virulence in a model that more closely mimics the clinical condition in humans was not clear. Since *B. cenocepacia* is a prominent pathogen in CGD, causing serious and deadly pneumonia, we used a lung infection in a CGD mouse (gp91^phox−/−^) and delivered bacteria via the intratracheal route (22). This is the same mouse reported previously by Sousa et al. (31); however, our method of administration of the bacteria used a non-surgical intratracheal delivery, while Sousa et al. (31) used a mid-line cervical incision to deliver bacteria to the trachea. They found that CGD mice given 10^3^ CFU of J2315 showed 100% mortality within 3 days (31). This was similar to what we observed in our initial infection (Fig. 1A). While Sousa et al. (31) did not assess the virulence of K56-2 in this model, they did test H111; mice infected with H111 also showed 100% mortality, but this took 6 days to be observed. Since we had previously noted H111 was non-pigmented (and has an R at position 378 of HmgA) (15), we hypothesized that expression of pigment might be associated with increased virulence. To the best of our knowledge, the virulence of J2315 and K56-2 has not been directly assessed and compared any murine model of respiratory infection. Here, we took advantage of the two ET-12 isolates, K56-2 and J2315, and confirmed that J2315 was more virulent than K56-2 (Fig. 1A).

In order to test whether the difference in virulence between J2315 and K56-2 in the CGD mouse was attributable to the function of HmgA, we generated isogenic strains where the *hmgA* gene from one strain was exchanged with the other; as anticipated J2315::K*hmgA* was non-pigmented, while K56-2::J*hmgA* was pigmented. Similar to our previous observation (15), we saw that J2315 and K56-2::J*hmgA* produced more pyomelanin when cultures were supplemented with 0.02% tyrosine, suggesting that pyomelanin production occurs through the tyrosine assimilation pathway, similar to other bacterial species. Furthermore, neither J2315::K*hmgA* nor K56-2 produced pigment even when cultures were supplemented with tyrosine.

Surprisingly, we observed no difference in the virulence of either of the strains vs their isogenic mutants in the CDG mouse model. This is distinctly different from what was observed in studying an *hmgA* deletion mutant in *Pseudomonas aeruginosa* PAO1 in CD1 mice: the PAO1 ∆*hmgA* strain made pyomelanin, and it was found that this pigmented strain was more virulent following intratracheal infection (32). Similarly, an *hppD* deletion mutant of the pigmented *Bordetella parapertussis* resulted in a non-pigmented mutant that was less able to colonize the lungs of C57BL/6J mice after intranasal infection (19).

In testing the susceptibility to reactive oxygen and nitrogen species, we observed a subtle difference in the survival of J2315 vs J2315::K*hmgA* in the presence of either H_2_O_2_ or SNP. This is different from what was observed by Keith et al. with an *hppD* mutant of the pigmented strain C5424 (13). They observed that the non-pigmented C5424 mutant was more sensitive to H_2_O_2_ compared to the wild-type pigmented parent. More in keeping with the results of Keith et al., we did observe that the pigmented version of K56-2, K56-2::J*hmgA*, showed increased resistance to H_2_O_2_ (at 12.5 mM) and some resistance to SNP (at levels below 12.5 mM). Altogether these results suggest that the importance of pigmentation for virulence and resistance to reactive oxygen and nitrogen species may be dependent on the bacterial species and/or strain background.

In order to determine what genes might be responsible for the different virulence of J2315 and K56-2, we compared the genomes of these strains using Rapid Annotation using Subsystem Technology. We found that J2315 encodes over 700 unique predicted open reading frames. Of particular interest is an error prone lesion bypass DNA polymerase V (UmuC) that is annotated in J2315 but does not appear to be present in the K56-2 genome. In *E. coli*, UmuC is a known SOS-induced repair polymerase that can respond to, and repair, damaged DNA (33). This type of repair polymerase could potentially contribute to the increased protection that J2315 exhibits to NO compared to K56-2. The impact of UmuC on the pathogenesis of J2315 vs K56-2 would require additional experiments and is the topic of future investigations.

Previous studies have examined the transcriptional impact of reactive oxygen treatment to J2315, and they reported an increased expression of genes related to oxidative stress response via the transcriptional regulator OxyR within 30 minutes of H_2_O_2_ treatment (34). The J2315 genome specifically encodes the *katB* gene, encoding catalase, which can breakdown hydrogen peroxide; this gene is absent from K56-2 (25, 35). The presence of factors that protect against oxidative stress may be contributing to the higher resistance of J2315 compared to K56-2 that we have observed in our experiments.

One limitation of the CGD mouse model is that, while this mouse is deficient in producing reactive oxygen species through NADPH, it is still capable of producing reactive nitrogen species, such as NO. While it might be suggested that mice defective in the ability to produce NO (iNOS^−/−^) would uncover the potential role of the pyomelanin pigment in the absence of reactive nitrogen species and presence of reactive oxygen species (36), previous studies with these iNOS^−/−^ mice showed that they were as resistant as wild-type mice to Bcc infection (36, 37).

In conclusion, the presence of pyomelanin is generally recognized for its role in resistance to reactive compounds produced as a host response to infection (38). Thus, the detection of pyomelanin-producing pathogens from clinical and environmental samples may be concerning (39). However, our data suggest that pyomelanin alone may not always constitute a virulence factor.
